# Diagnosis and Management of Barrett’s Esophagus

**DOI:** 10.3390/jcm12062141

**Published:** 2023-03-09

**Authors:** Maja Mejza, Ewa Małecka-Wojciesko

**Affiliations:** Department of Digestive Tract Diseases, Medical University, 90-153 Lodz, Poland

**Keywords:** Barrett’s esophagus (BE), esophageal adenocarcinoma, intestinal metaplasia (IM), GERD

## Abstract

Barrett’s esophagus is a metaplastic change of esophageal mucosa, which can be characterized by its salmon-colored lining and the presence of columnar epithelium with goblet cells. It is a well-established precancerous state of esophageal adenocarcinoma, a tumor with very poor survival rates, which incidence is rapidly growing. Despite numerous research, the debate about its diagnosis and management is still ongoing. This article aims to provide an overview of the current recommendations and new discoveries regarding the subject.

## 1. Introduction

Barrett’s esophagus (BE) is a condition that can be defined as a metaplastic change of esophageal mucosa characterized by its salmon-colored lining that ranges at least 1 cm proximal to the gastroesophageal junction (GEJ) [1]. It is most likely caused by the atypical healing process caused by esophageal injury [2]. The clinical importance of this disorder should not be underestimated, given that it is an established precancerous state of esophageal adenocarcinoma (EAC), a tumor with very poor survival rates [3] of very rapidly growing incidence [4,5,6]. Furthermore, cancers of the esophagus rank seventh in terms of incidence and sixth in mortality worldwide, meaning that they were the cause of one in every 18 cancer deaths in 2020 [7].

Estimations on the general prevalence of BE vary from 1.6 to 6.8% [8] in the general US population. However, such approximations are not very reliable due to numerous limitations, such as bias toward older patients, excluding BE < 3 cm, based on coding for BE dx (not pathology), adjusted for an increase in the use of endoscopy, performing the study in an at-risk population or only in symptomatic patients [8]. Data from European countries seem to be more consistent. A multicenter study from Southern European countries reported the overall BE prevalence to be 1.29% (95% CI: 0.73–1.85) [9], and in the general Swedish population, BE was present in 1.6% (95% CI: 0.8–2.4) of subjects [10]. The nationwide registry of pathology reports from the Netherlands suggests an increased incidence of both BE and EAC [11]. Incidence of BE increased by 41% (95% CI: 38–44%) among men and by 23% (95% CI: 19–26%) among women in 2000–2003, compared with 1992–1995. Similarly, between 1993–1997 and 2002–2005, there was a 159% increase in BE incidence in Northern Ireland population [12]. However, both studies were prone to be affected by increased endoscopy and biopsy rates during that period. These data imply that trends similar to the ones observed in the USA occur as well in Europe.

The purpose of this article is to review the latest literature regarding the subject and therefore help the physician to make informed decisions based on the most recent findings.

## 2. BE Risk Factors

Well-established risk factors, listed in the guidelines of the American Gastroenterological Association (AGA) [13] for this condition include male sex [14,15], white race [15], history of smoking [16,17,18], chronic gastroesophageal reflux disease (GERD) [16,19], obesity [16,20,21,22] and family history of BE or EAC [23,24]. Similar risk factors have been published in the Asia-Pacific consensus on the management of GERD [25]. Moreover, BE risk increases with age [26]. Additional risk factors mentioned in different meta-analyses include alcohol consumption [27], history of diabetes mellitus (DM), and oral non-metformin anti-diabetic medications [28]. A summary of all the BE risk factors can be found in Table 1.

Obesity is, as already mentioned, a significant risk factor for developing BE. However, it is important to add that most central obesity accounts for this phenomenon [21,22]. Apart from an association between abdominal obesity and GERD, there appears to be a separate one between larger waist circumference and BE [20,21].

Multiple predictive tools have been discovered in order to help clinicians decide who should undergo screening or surveillance. Methods that prove to be more effective than GERD alone include HUNT, M-BERET and Kunzmann [29]. HUNT (Nord-Trøndelag health study) included risk factors for EAC such as male sex, older age, GERD in the prior 12 months, obesity and history of tobacco smoking [30]. M-BERET (Michigan Barrett’s esophagus prediction tool) was based on a population of men aged 50–79 who presented for colonoscopy in order to have screening for colorectal cancer [31]. The model is developed from a questionnaire during which the patients were asked about their history of smoking and GERD symptoms. Moreover, the age and measured waist-to-hip ratio of the patients were considered [31]. Lastly, the Kunzmann tool was based on individuals (all of who were older than 50 years) enrolled in the UK Biobank prospective cohort study [32]. Similarly to the previous tools, age, sex, smoking history, BMI and history of esophageal conditions, such as GERD BE, as well as hiatal hernia, esophageal stricture, undergone fundoplication or the use of acid-reducing medications were considered [32].

The goal of diagnosing and further screening in BE patients is early detection of dysplasia and EAC. According to a multicenter U.S. study [5] the majority of patients diagnosed with BE do not have dysplasia (70.1% non-dysplastic BE vs. 16.9% low-grade dysplasia and high-grade dysplasia). Appropriate management and surveillance may reduce the morbidity and mortality associated with EAC.

## 3. BE Diagnosis

To establish the diagnosis, the histopathological confirmation of intestinal metaplasia (IM), defined by the presence of columnar epithelium containing the goblet cells, is necessary, according to the majority of gastroenterological societies, except for the British Society of Gastroenterology [33]. While describing the BE, the experts suggest using Prague and Paris classifications. Prague classification is a method of describing the length of BE segment at endoscopy. It considers both the circumferential extent (C) and maximum length (M) of metaplasia and was proven to be effective in describing BE [34]. Circumferential length is measured as the distance between the GEJ and the proximal margin of the circumferential BE extent. Maximum length is the distance between the GEJ and the proximal margin of the longest tongue [35]. Importantly, even if the BE “islands” are present more proximally, only the continuous extent should be taken into the measurement of the M parameter [35,36]. Paris classification is used afterward to describe any visible lesion. In this classification superficial neoplastic lesions are divided into polypoid (0-Ip–pedunculated, 0-Is–sessile) or non-polypoid (0-IIa–slightly elevated, 0-IIb–flat, 0-IIc–slightly depressed, 0-III–excavated (ulcer)) [37]. “Superficial” in this terminology means that the lesion does not infiltrate the submucosa.

One of the most promising novel methods for BE diagnosis is a Cytosponge—a gelatin capsule attached to a string. The capsule is meant to dissolve 5 min after being swallowed, enabling the device hidden inside to expand. Afterward, the apparatus is retrieved by pulling on the string, and as it passes through the esophagus, it collects the tissue specimen [38,39]. The sampling is then analyzed using immunostaining for g for trefoil factor 3, a diagnostic marker of BE [40]. Cytosponge was proven to be more cost-effective than endoscopy due to its higher uptake [40].

## 4. BE Risk of Progression to EAC

Non-dysplastic BE (NDBE) is diagnosed when there is an IM without any histological evidence of dysplasia. Most recent analyses show that NBDE to EAC annual progression rates are between 0.33 and 0.70% [41,42,43,44,45,46]. Desai et al. [42] suggest that the former reports on higher incidences resulted from methodical errors. Shaheen et al. [47] showed an inverse relationship between study size and reported cancer risk, supporting the opinion that the real progression rate is most likely closer to the lower end of this range. A study published in 2011 excluded the patients who developed EAC within the first year after NDBE diagnosis and found an even lower rate of 0.12% [48]. It has also been established that patients with two endoscopies showing NDBE, compared with those who only received the NBDE diagnosis once, were almost four times less likely to obtain an EAC diagnosis on their next endoscopy [49]. Another factor affecting these results is the length of the BE segment. Short segment BE (<3 cm) progression rates were 0.06% (95%CI: 0.01–0.10%) in comparison with 0.31% (95%CI: 0.21–0.40%) for long segment BE (≥3 cm) [50]. Despite the low absolute risk, the relative risk (RR) is high when compared to the general population. Due to this fact, most leading gastroenterology societies recommend an NDBE endoscopic surveillance every 3–5 years [13,51,52]. Regular surveillance surely leads to an earlier detection of dysplasia, although it is yet to be proven whether it is beneficial for the patient’s survival. A case-control study from 2013 [53] showed that 3 years of surveillance was not associated with decreased risk of death from esophageal adenocarcinoma (adjusted OR: 0.99; 95%CI: 0.36–2.75). However, there are also studies showing the reduced EAC-related mortality in patients undergoing regular surveillance [54]. There are numerous reasons that can explain the disparity between those results—one of them being the differences in surveillance biopsy protocols around the globe.

Low-grade dysplasia (LGD) is defined by pathologists as the cellular atypia with co-occurring preservation of glandular architecture. In LGD, the rate of progression to EAC or HGD fluctuates between 0.4 and 13.4% [41,48,55,56,57,58,59,60]. This disparity is most likely due to overdiagnosis of LGD, meaning that a large proportion of LGD cases ought to be downstaged to NDBE [55,58]. Differences in the classification of LGD by pathologists are presumably accounting for this uncertainty [55], making the creation of adequate protocols for LGD management problematic.

High-grade dysplasia (HGD) is a synonym of carcinoma in situ, meaning that the neoplasia does not extend the basement membrane. It is, therefore, associated with the highest, i.e., 59% (95% CI: 44–75) cancer incidence within 5 years [61]. The uniquity of esophageal lymphatic vessels is that they can frequently extend into the mucosa, making metastasis possible even in the early phase of a malignancy [62]. Nevertheless, the systematic review regarding the risk of lymph node metastases in HGD and EAC detected no such metastases in HGD [63]. Data on metastases in HGD is scarce. Even metastases in EAC are not frequently detected. The aforementioned study found that only 1.93% (26/1350) of patients with a diagnosis of intramucosal adenocarcinoma had positive lymph nodes [63]. Nonetheless, poor interobserver agreement on the differentiation between HGD and EAC has been reported [64] by pathologists. Therefore, caution should be advised in the interpretation of those data.

Several molecular markers were found to be useful in identifying patients with an increased risk of malignancy. The most widely studied genetic alterations in NDBE are of p53 and p16—two tumor suppression genes [65]. The frequency of p16 mutations is not high, although it is found to be rising in BE–dysplasia sequence [66], which means it can contribute to the early phase of progression from BE to EAC. Importantly, only a minority of tumors progress in a manner of stepwise accumulation of tumor suppressor mutations. One study found that 62,5% of EAC develop through a genome doubling of p53-mutant cells [67]. This may explain why a lack of p53 expression is considered a powerful predictor for neoplastic progression in patients with BE [68]. Another potential malignant progression marker is the APC gene. Its loss of function concerns a minority of patients with BE, yet it was shown to increase the risk of progression to EAC [69].

Before enrolling a patient in a surveillance program, clinicians need to make sure they explained all the risks, benefits and alternatives such as endoscopic eradication therapy (EET) or surgery. Studies have shown that increasing the inspection time is associated with increased detection of both EAC and high-grade dysplasia [70]. Lately, new diagnostic approaches have been developed to enhance rates of dysplasia detection during surveillance. Two of them are dye chromoendoscopy and virtual chromoendoscopy (VC). Several different dyes may be used in conventional chromoendoscopy to enhance the visibility of mucosal abnormalities. One of them—methylene blue (MB)—is not absorbed by normal squamous epithelium; however, metaplastic epithelium does absorb it, leading to easier identification of IM [71]. In comparison with the standard four quadrant biopsies (4QB), MB was shown to be similar in the rates of detection while requiring significantly fewer biopsies (18.92 ± 6.36 for 4QB in comparison with 9.23 ± 2.89 for MB (*p* < 0.001) [72]. However, another study has found 4QB to be significantly more sensitive at detecting dysplasia in BE than MB (*p* = 0.02) [73]. Moreover, a meta-analysis from 2009 did not find any benefit from the use of MB in comparison with white-light endoscopy with random biopsies for detecting dysplasia in BE [74]. Additional data suggest that MB could be associated with DNA damage [75]. However, it was not adequately proven yet. Moreover, a recent clinical trial found no detectable DNA damage was caused by oral intake of methylene blue with colonic delivery [76]. Acetic acid (AA), an alternative dye, exhibits high diagnostic accuracy for HGD/EAC detection. After its application, esophageal mucosa turns white and columnar epithelium turns reddish, which makes it easier to distinguish [71]. In one meta-analysis, the pooled sensitivity of AA-based diagnosis was 0.92 (95% CI: 0.83–0.97), and the pooled specificity was 0.96 (95% CI: 0.85–0.99) [77]. Furthermore, AA-guided protocols were found to be more effective than the Seattle protocol in a high-risk Barrett population. Using only the targeted AA biopsies in this high-risk population resulted in a 4% miss rate; however, it was proved to be even more cost-effective than the simultaneous use of random biopsies [78].

VC was developed to save time and lessen the technical inconvenience associated with dye chromoendoscopy [79]. In this procedure, the same effect is obtained without the use of additional equipment. One of the most widely studied systems is narrow-band imaging (NBI). The device utilizes a very specific wavelength to accentuate both vascular and mucosal patterns, making the detection of abnormalities easier for endoscopists [80]. It was shown to have some advantages over currently recommended, high-definition white light endoscopy with the Seattle protocol having the same IM detection rate, NBI requires fewer biopsies and can detect more areas with dysplasia [81]. Moreover, a study based on the data from NHS England proved NBI to be more cost-saving than the currently recommended technique [82]. Other currently studied VC systems are flexible spectral imaging color enhancement (FICE) and i-scan [83]. Targeted biopsies with the second system have significantly higher diagnostic yield for Barrett’s epithelium detection than random biopsies (63% vs. 24%; *p* = 0.0001). Moreover, the i-scan had a 96% (k = 0.92) accuracy for predicting the metaplasia [84]. Nonetheless, data on these systems in BE and related conditions are still scarce; therefore, their usefulness in clinical practice is yet to be established.

Magnification endoscopy is another technique that is used to enhance the detectability of pathologies. It is based on a system of moveable lenses, which allow for optical magnification [85]. Magnification enables the physician to see and analyze a characteristic structure called a pit pattern. The use of magnification endoscopy with the pit pattern analysis has higher sensitivity than the use of methylene blue chromoendoscopy [86]. Moreover, magnification endoscopy may correspond to an increase in cell cycles in BE [87].

## 5. EAC Screening in BE

Guidelines of different regions have variable approaches to the BE screening strategy (Table 2). Asia-Pacific consensus on the management of GERD pointed out that the low prevalence rates in this population make screening for BE in the region unnecessary [25]. Guidelines of the American Gastroenterological Association (AGA) [13] suggest that screening may be considered for those with at least three well-established risk factors for BE and EAC, such as the following: being male, non-Hispanic white, age >50 years, having a history of smoking, chronic gastroesophageal reflux disease, obesity, or a family history of BE or esophageal adenocarcinoma. Importantly, in those guidelines, chronic gastroesophageal reflux disease (GERD) is not considered a mandatory requirement to screen for BE. Despite being one of the most well-documented risk factors for BE, GERD was, in numerous pieces of research, the reason why most patients with EAC did not meet the screening guidelines [88,89]. The first of the mentioned studies [88] provides the comparison of sensitivity and specificity between the previous AGA guidelines (requiring two or more BE risk factors) and the other guidelines, which required GERD first. The AGA guidelines for BE screening had 100% sensitivity and 0.2% specificity, and the others had, respectively, 38.6–43.2% sensitivity and 67.4–76.5% specificity. Moreover, in this study, more than half of the patients diagnosed with BE were not experiencing GERD symptoms frequently, but nearly all of them had at least one known BE risk factor. In the latter study, 54.9% of EAC patients in the US and 38.9% in the UK were not identified when ACG or BSG guidelines were used as a criterium [89]. Among those, who did not meet screening criteria, only 13.5% in the US group and 38.6% in the UK one experienced heartburn. Elimination of chronic GERD from ACG/BSG guidelines improved qualification for screening from 45.1% to 81.3% (*p* < 0.001) in the US and from 61.1% to 81.5% (*p* < 0.001) in the UK. Therefore, the guidelines that consider GERD symptoms mandatory can lead to the underdiagnosis of some patients. However, GERD is a condition with a very high prevalence in Europe, its prevalence is estimated to be 8.8–25.9% [90], and consequently, the AGA criteria has virtually no specificity.

**Figure 1:**
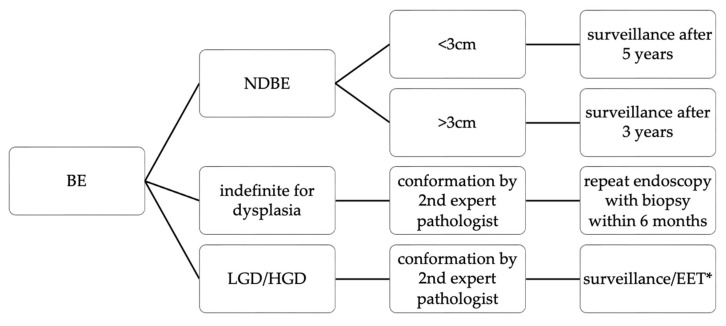
Care algorithm for patients with diagnosed BE-based on ACG guidelines [91]. * Precise recommendations on the management of LGD and HGD can be found in Table 3.

AGA recommends using high-definition white light endoscopy (HD-WLE) and virtual chromoendoscopy (VC) during screening and surveillance [13]. In patients with NDBE, surveillance ought to be performed every 3–5 years [13]. Guidelines of the American College of Gastroenterology (ACG) recommend basing the surveillance frequency on the length of BE segment (Figure 1) [91]. On the other hand, the international BOB CAT (Benign Barrett’s and Cancer Taskforce) consensus does not make any recommendations about surveillance in NDBE, except for stopping it in patients with <5 years of life expectancy [92].

Regular endoscopic surveillance of BE is widely accepted [13,52,91,93], despite sparse evidence. As previously explained, it is not clear whether surveillance programs decrease mortality from EAC [53,54,94]. Moreover, the interventions are costly and inconvenient for the patients. The economic models that were developed for determining the cost-effectiveness of different strategies for BE management are sensitive to assumptions about rates of progression, meaning that even small changes in these rates can result in significant alteration to their conclusions [95]. With a considerable variability in established rates of progression, there are not many things that we can be certain about when discussing the cost-effectiveness of surveillance. Surely, surveillance is the only recommended strategy for NDBE management [95] (Figure 1).

Major gastroenterological associations recommend using the Seattle biopsy protocol during both screening and surveillance [13,52]. In this protocol, four-quadrant biopsies are performed randomly every 1–2 cm in addition to targeted biopsies taken from visible lesions. It is shown to be more effective in IM detection than any other established method [96,97]. However, according to a meta-analysis from 2016, up to 25.3% (95% CI: 16.4–36.8%) of EACs were missed during surveillance and 23.9% (95% CI: 15.3–35.4) when the analysis was restricted to NDBE patients [98]. In addition to the Seattle protocol, AGA recommends using WATS–3D, a novel, commercially available method for sampling the suspected or established BE segment [13]; however, we still lack strong data proving its effectiveness in comparison to the protocol. WATS–3D (wide-angle transepithelial sampling with computer-assisted 3-dimensional tissue analysis) is a device that samples a wide circumferential surface using a brush and resects tissue samples [99]. Sampled cells are then analyzed using a computerized microscope and neural network, which create a 3D tissue model [99]. All the pathologies, such as IM, dysplasia and EAC, can be found in the model [99].

In recent years artificial intelligence (AI) has emerged as a promising tool that could improve the effectiveness of BE screening. One study from 2021 compared a fully automated deep learning algorithm for identifying BE with the manual human assessment [100]. The algorithm was developed in a neural network structure of fully convolutional networks (FCN). The first stage of the study included training the program with endoscopic images. Afterward, the program was tested with a different set of images. The performance of the algorithm was measured by intersection over union (IOU). IOU compared a localization of the GEJ/SCJ (gastroesophageal junction/squamous-columnar junction) predicted by the AI with its true localization. The average IOU values were 0.56 for the GEJ and 0.82 for the SCJ, respectively, which indicated its possible usefulness in the future.

In 2022 a meta-analysis examined the use of AI in surveillance for BE-related neoplasia [101]. It has shown the pooled sensitivity, specificity and diagnostic odds ratio to be 90.3% (95% CI: 87.1–92.7%), 84.4% (95% CI: 80.2–87.9%) and 48.1 (95% CI: 28.4–81.5) respectively. However, this analysis has a very important limitation, which is that the analyzed studies do not include LGD. Other mentioned limitations were the fact that the included studies were retrospective; some of them only included <100 patients in their training model, and there was not enough data to compare the results to the performance of expert endoscopists. In spite of that, it surely displays the need for prospective studies for further investigation into the effectiveness of these techniques.

Studies have recommended numerous endoscopy techniques that can increase the likelihood of identifying subtle abnormalities, such as through irrigation of mucosa, and adequate time spent for examination using HD-WLE [70]. ESGE recommends ≥7 min for upper endoscopy and ≥1 min for every cm of the circumferential extent of metaplasia. It is also crucial to pay additional attention to the right hemisphere of the esophagus as it is the area where EAC has a predilection to develop [102].

## 6. BE Management

### 6.1. Endoscopic Ablation Therapy

The most widely used method is radiofrequency ablation (RFA). It uses high-power thermal energy generated by radiofrequency to promote necrosis in the remaining IM [103]. Importantly, its penetration depth allows for the reaching both the epithelial and muscularis mucosa layers without profoundly damaging the submucosa [103]. Despite generally being considered a safe procedure, RFA account for adverse events, including esophageal strictures, perforations, hemorrhages and chest pain.

In the case of LGD British Society of Gastroenterology (BSG), similarly to the Polish guidelines [94], recommends confirmation of the diagnosis by two expert pathologists and endoscopic ablative therapy, preferably with RFA, as a management strategy [104]. ACG, on the other hand, recommends discussing both risks and benefits of EET vs. surveillance and recognizes both methods as recommended [91]. The latter also emphasizes the fact that there is little evidence of different management strategies in LGD; therefore, the best management of LGD is likely yet to be found. Not all guidelines agree in recommending ablation as an optimal strategy for LGD management (Table 3).

When it comes to comparison between RFA and surveillance, available data suggest that RFA use results in a significant reduction in risk of progression to HGD/EAC (RR = 0.14%; 95% CI: 0.04–0.45; *p* = 0.001) [105]. This result was supported by a later analysis [106]. Contrastingly, one study found no significant reduction in progression to HGD or EAC in the RFA cohort compared to surveillance [107]. Consequently, there is still room for further investigation into the optimal strategy for LGD treatment.

Endoscopic ablative therapy has also been recommended for patients with HGD and T1a EAC after endoscopic eradication therapy (EET) [91]. In one study, complete eradication occurred in 81% of the patients with HGD treated with RFA, in comparison with 19% in the group that underwent a sham procedure (*p* < 0.001). Furthermore, 19% of patients in the control group progressed to EAC. Contrastingly, in the ablation group, progression occurred only in 2.4% of patients (*p* = 0.04) [108]. The reason for ablation in the case of T1a EAC after EET is to reduce the risk of recurrent dysplasia/EAC [91]. One study compared the recurrence rate in patients who underwent ablation to the surveillance-only group. Only 1 secondary lesion (3%) was detected in the ablation group, compared with 11 (36.7%) in the surveillance group [109].

**Table 3:** Recommended management of LGD and HGD.

	LGD	HGD
Polish guidelines [93]	Endoscopic ablation therapy (preferably with RFA), surveillance after 6 months if ablation was not undertaken (recommendation grade A for ablation and C for surveillance)	Endoscopic therapy (options ought to be discussed by an upper GI multidisciplinary team beforehand)
BSG [33,104]		
ESGE [52]	Endoscopic ablation therapy (with RFA)	Endoscopic resection is recommended for all the visible lesions. If no lesions suspicious for dysplasia are visible, endoscopist ought to take 4-quadrant biopsies. In case of histopathological conformation of the HGD, endoscopic ablation (with RFA) is recommended. Otherwise, endoscopy should be repeated after 3 months.
AGA [110]	Surveillance or EET (endoscopic eradication therapy)	EET (removal of visible lesions with EMR)
Australian [111]	Surveillance every 6 months, however, reverting to a less frequent follow-up schedule should be considered if two consecutive endoscopies show no dysplasia or Endoscopic ablation may be considered—especially if LGD is definite, multifocal and present on more than one occasion	Endoscopic resection of visible/nodular lesions and RFA within the flat segments
ACG [91]	Surveillance (6 and 12 months after diagnosis, and annually thereafter) or EET (resection of all visible lesions, followed by ablation) and then surveillance endoscopy after 1 years, 3 years and afterwards continued every 2 years)	EET (resection of visible lesions followed by ablation and surveillance 3, 6 and 12 months after complete eradication of remaining metaplastic epithelium and then continued annually)
Asia-Pacific consensus [25]	Surveillance endoscopy after 6 months or Endoscopic resection of focal lesions and if there is the absence of such lesions consider RFA	Endoscopic resection followed by RFA

### 6.2. Endoscopic Eradication Therapy

EET is a standard procedure for BE-related neoplasia management. It is the resection of all lesions visible during an endoscopy, followed by ablation of the remaining IM to minimize the risk of metachronous dysplasia. This method is recommended by both European and American guidelines in HGD treatment [52,91,110]. Comparative effectiveness of esophagectomy vs. EET for HGD proves EET to be better for all age groups [112]. More precisely, for a 65-year-old patient, EET-RFA yields equivalent utility (11.47 vs. 11.44 dQALY for respectively EET-RFA and esophagectomy). In this analysis, dQALY is used instead of QALY, which means that all costs and utilities were discounted at an annual rate of 3%. Additionally, EET-RFA was shown to have lower costs (USD 52.5 K vs. USD 74.3 K) over the first 20 years. The dominance of EET-RFA was moreover shown to dominate both surveillance and esophagectomy in all patients age groups. The groups of patients for which surgery turned out to be more effective were those with diffused or ulcerated HGD.

Another stage for which EET is a recommended strategy is T1a EAC [52,91]. In an analysis from 2018, esophagectomy provided more unadjusted life years than endoscopic therapy (6.97 vs. 6.81) but fewer quality-adjusted life years (QALYs, 4.95 vs. 5.22) [113]. Furthermore, in this analysis esophagectomy cost USD 34,834.35 more. Therefore, it confirmed the current recommendations. Additionally, early (T0 or T1a) esophageal cancer-related mortality did not differ between EET and esophagectomy (*p* = 0.22) [114]. However, there was a significant difference in mortality related to other causes (predominantly heart disease) (EET: 13.5% vs. esophagectomy: 5.7%, *p* < 0.001).

There are the following two standard resection techniques: endoscopic mucosal resection (EMR) and endoscopic submucosal dissection (ESD). The major difference between them is that ESD allows for the resection of a larger area [115]. EMR is performed using band ligation or cap suctioning. It is considered an older technique, and it has a higher risk of local recurrence than ESD, especially when lesions are larger than 15 mm [116]. It may be due to the fact that using this technique, larger lesions (>20 mm) are often removed piecemeal [117], which increases the risk of local recurrence [118] and may make the pathologic assessment of resection more challenging [119]. This, among other reasons, made ESD the standard treatment in East Asian countries [120]. Moreover, ESGE also recommends the use of ESD for lesions that are suspicious of invading the submucosa, for malignant lesions that are above 20 mm, and for lesions in scarred or fibrotic areas [121]. In case of smaller lesions that are less likely to invade the submucosa or large/multifocal benign lesions, ESGE recommends using EMR.

However, complications connected with ESD must be acknowledged. When treating superficial esophageal carcinoma, the pooled incidence of stenosis was 5% (95% CI 3–8%) and of perforation–1% (95% CI 0–1) [122]. Other authors compared ESD to EMR and found that ESD was associated with higher rates of perforation (4.0 vs. 1.3%) [123]. Several methods for closing the perforation have been discovered to prevent further damage associated with this complication. AGA advises either the use of clip closure or endoscopic suturing for perforations caused by ESD [124]. Clips used in this procedure can be divided into the following two groups: through-the-scope clips (TTSCs) and over-the-scope clips (OTSCs) [125]. The latter has the advantage of the ability to close the leakage even if the surrounding tissue is inflamed or endured [126]. Endoscopic suturing allows for the closure of larger defects; however, this technique is much harder than clip application [126]. Other possible closure techniques include stent placement, endoscopic vacuum-assisted closure and the use of tissue sealants [126].

Results from the Dutch database clearly show both the short and long-term efficacy of EET, followed by RFA, as well as the incidence of complications [127]. In this study, complete endoscopic eradication of all visible BE was archived in 94% (95% CI 93 to 95) of patients (1270/1348) with LGD, HGD or low-risk EAC. The most common complication was esophageal stenosis—it occurred in 15% of patients (95% CI 13 to 17). Out of 210 patients who developed stenosis, 40 required more than 5 (median 9) endoscopic dilatations. Moreover, 10 of these patients required additional incision therapy and 4 esophageal stent placements. Risk factors for this complication included BE length, prior ER and more extensive prior ER. Perforations occurred only after ER or endoscopic dilatation of stenosis, and their incidence was 1% (95% CI 0 to 1). Recurrence of dysplasia or EAC during follow-up occurred in 3% of patients (annual risk 1.0% (95% CI 0.8 to 1.4)), and 0.7% of patients had a worse disease stage than at baseline staging. During a median follow-up for vital status after 60 months (p25-p75 38–86) after baseline or 49 months (p25-p75 26–72), after the last treatment 96 patients died, but only 4 of those deaths were due to metastasized EAC, while the rest was due to unrelated causes.

Recurrence rates per patient year after complete eradication of BE are 8–10% for IM and 2–3% for dysplasia [128]. These rates establish that it is crucial for physicians to understand the subtle changes in endoscopic images after EET and RFA. Importantly, Yang et al. [129] recently described two endoscopic features of sub-squamous intestinal metaplasia (SSIM), meaning IM located under the normal squamous epithelium. This can develop with or without prior endoscopic treatment; however, this is especially important in post-EET surveillance. These features included darker pink or darker brown mucosa underneath squamous epithelium and raised areas underneath squamous mucosa. At least one of these features was present in 79% of patients with a histology-confirmed disease [129]. Another study argued that discoloration could occur in numerous areas of the post-RFA esophagus, and finding prominent subepithelial glandular structures with volumetric laser endomicroscopy was more effective in locating the SSIM [130]. This study also established differences in endoscopic findings between developing squamous mucosa and regenerating residual BE. In white light endoscopy, both can appear erythematous, but BE has a more uneven texture and no white exudate. In NBI, the most evident sign of regenerating BE was the emerging pit pattern.

### 6.3. Surgery

EET techniques have become the preferred management strategy for HGD and early EAC, given that esophagectomy is perceived as a high-risk procedure and it is less cost-effective across all the age groups for treating HGD and T1a cancers across all age groups [112,113]. However, when treating T1b EAC, age and comorbidities account for alteration in the choice of the optimal treatment strategy [113]. Esophagectomy is more cost-effective than EET but only for healthy patients with T1b EAC younger than 70 (for patients aged 70 at the diagnosis, incremental cost-effectiveness ratio (ICER) was equal to USD 96,630) [113]. In the case of older patients with more comorbidities, esophagectomy provided more unadjusted and quality-adjusted life years but was not cost effective (ICER equal to USD 156,980.91). When it comes to locally advanced tumors, the best treatment is esophagectomy with the support of preoperative chemotherapy [131]. However, in the case the patient is unwilling or unsuitable for the surgery, definitive chemotherapy alone is considered equally effective [132]. In advanced stages, optimal management ought to be palliation.

## 7. BE Prevention

Dietary fiber intake was inversely associated with the risk of Barrett’s esophagus and esophageal cancer [133]; however, this meta-analysis should be treated with caution, considering the limited number of studies included and their heterogeneity. Moreover, the influence of other potential confounders might not have been considered. Similarly, a former meta-analysis from 2013 suggests an inverse association between dietary fiber intake and EAC carcinogenesis (OR = 0.66; 95%CI: 0.44–0.98); however, its authors likewise noticed heterogeneity (I^2^ = 83%; *p* < 0.001) in the analyzed studies [134]. Importantly, the differences likely influence only the magnitude of the protective effect. Therefore, increasing dietary fiber intake is most likely a good recommendation for people at risk of developing EAC. The authors of the latter study suggested that the effect may be caused by the inositol hexaphosphate, which is present in high-fiber foods [134]. This substance was shown to inhibit the proliferation of esophageal adenocarcinoma cells in vitro [135]. Another mechanism that may explain this phenomenon is that increased fiber intake is associated with a lower frequency of refluxes and increased lower esophageal sphincter (LES) minimal resting pressure [136].

Lifestyle changes were proven to reduce the risk of BE [137] as well as GERD [138]. The inverse association was found between BE risk and the higher intake of vitamin C (RR = 0.59; 95% CI: 0.44–0.80), folate (RR = 0.47; 95% CI: 0.31–0.71) and fiber (RR = 0.95; 95% CI: 0.93–0.97). The previous study by the same author observed a similar association between BE risk and vegetable intake (OR = 0.45; 95% CI: 0.29–0.71) [139]. One large Dutch study found that a one-point increment of the healthy lifestyle score (HLS), which combined nonsmoking, having a normal BMI, being physically active and adhering to a Mediterranean diet with no or low alcohol intake, was associated with an HR reduction of 31% for esophageal cancer [140]. Nevertheless, for EAC, association with HLS was not significantly inverse. Another cohort study examined the connections between the risk of EAC and two diet quality indices, the Healthy Eating Index–2005 (HEI–2005) and the alternate Mediterranean diet score (aMED) [141]. No significant association between aMED and EAC was observed after adjusting for covariates, such as smoking; however, higher HEI–2005 scores were associated with significantly reduced risk of EAC (HR = 0.75, 95% CI 0.57–0.98). The first conclusion was supported by another cohort study from the Netherlands, which also have not found an association between Mediterranean diet adherence and EAC risk [142]. It is important to remember that in such analyses, it is difficult, if not impossible, to exclude all possible covariates. However, despite established contradictions, some dietary interventions can most likely decrease the risk of BE and related neoplasia.

Smoking is associated with an increased risk of BE [17,18]. Moreover, the risk of BE increases with higher pack years of cigarette smoking (*p*-trend < 0.01) [143]. Furthermore, the risk of BE decreases with the increasing duration of smoking cessation (*p*-trend = 0.01) [143]. Therefore, smoking cessation programs could prove to be useful in reducing the pathologies connected to BE.

Alcohol consumption and BE risk data vary in the literature. It was shown that those who reported consuming 3–<5 drinks per day had a statistically significant lower risk of BE than non-drinkers (OR = 0.57, 95% CI 0.38–0.86) [144]. Interestingly, a meta-analysis of observational studies from 2015 found an association between alcohol consumption and increased risk of BE but only in men (RR = 1.35, 96% CI 1.13–1.61, I^2^ = 0.00%) and the Asian population (RR = 1.60, 95% CI 1.03–2.49, I^2^ = 60.60%) [145]. Moreover, alcohol consumption was not associated with neoplastic progression to EAC [146]. Therefore, it is not clear whether decreasing alcohol consumption in the general population may decrease the incidence of BE, and prospective studies are needed in order to clarify this issue [145].

The use of proton pump inhibitors (PPI) could be another potential defense against progressing from BE to HGD or EAC [147,148]. Despite potential drawbacks, such as PPI-associated adverse effects (PAAEs), PPIs are evidently reducing the risk of EAC [149]. One meta-analysis established that PPI use was connected with a 71% decrease in risk of EAC or/and HGD (the adjusted OR = 0.29; 95% CI: 0.12–0.79) [150]. Moreover, another study found that there is a significant difference between the intake of high-dose and low-dose PPI (time ratio for EAC was equal to 1.04 (95%CI: 0.67–1.61) and for HGD 1.36 (0.92–2.02) [148]. Furthermore, in this, the number needed to treat (NNT) was 34 for high-dose PPI. When considering the best option for the patients, it is important to note that anti-reflux surgery is not significantly superior to PPI therapy in regard to preventing BE from progressing to EAC [151,152].

NSAIDs, particularly aspirin, were associated with a reduced risk of BE [153,154]. Importantly, the first of the mentioned studies [153] proved people with BE were less likely to use aspirin than the control group (OR = 0.59; 95% CI: 0.39–0.87). This inverse connection was even stronger when the control group consisted of patients with GERD symptoms (OR = 0.49; 95% CI: 0.32–0.75; *p*-value interaction term for GERD symptoms = 0.004). Additionally, moderate and high total aspirin intake was associated with lower risk of BE (at least weekly use for less than 5 years (moderate total intake): OR = 0.41; 95% CI: 0.23–0.73; for weekly, or more often use for over 5 years (high total intake): OR = 0.46; 95% CI: 0.26–0.79). The second study [154], a meta-analysis, demonstrated that NSAIDs could reduce BE risk (OR = 0.84; 95% CI: 0.75–0.94, *p* < 0.05). However, when subgroup analysis was performed, this relation was only evident in females. Moreover, NSAIDs may even act after the formation of BE in the progression toward EAC [148,155]. In this meta-analysis, aspirin and other NSAIDs, the use was associated with the reduction of EAC risk in patients previously diagnosed with BE (RR = 0.64; 95% CI: 0.42–0.96), as well as in the general population (for aspirin: OR = 0.73; 95% CI: 0.65–0.83 and for other NSAIDs: OR = 0.84; 95% CI: 0.72–0.98). The clear dose-effect relationship and its constitutive mechanisms are yet to be fully established. Some studies do not confirm the association between NSAIDs and BE risk [156,157]; thus, there is not enough data to support the use of these drugs to reduce the risk of BE.

In a 2020 study, selective serotonin reuptake inhibitors (SSRI) usage trended strongly towards significance in protecting (*p* = 0.08) from progression to EAC [28]. The mechanism is not well-understood; however, SSRI decreases levels of insulin-like growth factor (IGF), and activation of the IGF–1 pathway in BE may play a role in the progression to EAC [158]. The common use of those drugs in recent years may point to further investigation into this potential benefit.

Other drugs associated with a reduction in BE progression to EAC are vitamin D or supplemental calcium [28]. Barrett’s mucosa, unlike normal squamous epithelium, has shown immunopositivity for the vitamin D receptor (VDR) [159], which may indicate increased sensitivity to the effects of this micronutrient; however, the exact mechanism is yet to be explored. The potential anticancer effects of vitamin D have been explored for many decades [160,161]. The biologically active form of vitamin D—calcitriol—is the substance that binds to the VDR. After binding to yet another receptor, the complex can affect gene transcription and translation by binding to its vitamin D response element [162]. Whether vitamin D status could decrease the risk of BE and EAC is not yet clarified [163]. Thus, further research is needed in order to establish both the association and the possible mechanism.

Zhang et al. [164] demonstrated that acidic bile salts can induce epithelial-to-mesenchymal transition (EMT) of Barrett’s cells. EMT is the process through which epithelial cells acquire mesenchymal cell characteristics, such as the ability to migrate. The conversion of epithelial cells into motile cells may underlie the pathogenesis of SSIM. Therefore, reducing exposure to these salts may prevent recurrent Barrett’s metaplasia after RFA.

## 8. Conclusions

Even though BE is a known condition, its diagnosis and management need improvement. The biggest challenge is the diagnosis and treatment of LGD. Data on its incidence and progression is not coherent. Therefore, strong evidence for the best management guidelines is yet to be found. Certainly, when it comes to HGD and most EAC cases, the best intervention is EET, followed by ablation, and in the case of NDBE, it is surveillance. Debate on LGD management is still ongoing; however, Polish, British and European guidelines consider endoscopic ablation therapy, while others recommend surveillance. Surgery is generally not recommended for EAC treatment; however, in younger patients with T1b EAC, it is more cost-effective than EET. There are numerous factors that could potentially decrease the risk of developing BE; however, data on which intervention ought to be proposed to patients is still scarce. Although great progress in BE pathology, pathophysiology, diagnostics and management was noted in the last decades, many questions remain unanswered. New research is necessary before we will be able to clearly reassure our BE patients of their worries and uncertainties.

## Figures and Tables

**Table 1:** Risk factors for Barrett’s esophagus.

Risk Factor	Mentioned in
Male sex	[13,14,15]
White race	[13,14,15]
History of smoking	[13,16,17,18]
Chronic GERD	[13,16,19]
Obesity	[13,16,20,21,22]
Family history of BE/EAC	[13,23,24]
Age > 50	[13,25]
Alcohol consumption	[27]
History of DM and oral non-metformin Anti-diabetic medications	[28]

**Table 2:** Criteria for BE screening.

Association	Criteria	Risk Factors
AGA [13]	≥3 risk factors	- age >50 years - male sex - non-Hispanic white - history of smoking - chronic GERD - obesity - family history of BE or esophageal adenocarcinoma.
ACG [91]	Chronic GERD and ≥3 risk factors	- age >50 years - male sex - white race - tobacco smoking - obesity - family history of BE or EAC in a first-degree relative.
BSG [33]	Chronic GERD and ≥3 risk factors (the threshold of multiple risk factors should be lowered in the presence at least one first-degree relative with BE or EAC)	- age ≥ 50 years - white race - male sex - obesity
ESGE [52]	Screening for BE is not advised but can be considered in patients with GERD >5 years and multiple risk factors	- age ≥ 50 years - white race - male sex - obesity - first-degree relative with BE or EAC.

## Data Availability

No new data were created or analyzed in this study.

## References

[1] Shaheen N.J., Falk G.W., Iyer P.G., Gerson L.B. (2016). ACG Clinical Guideline: Diagnosis and Management of Barrett’s Esophagus. Am. J. Gastroenterol..

[2] Wang R.-H. (2015). From reflux esophagitis to Barrett’s esophagus and esophageal adenocarcinoma. World J. Gastroenterol..

[3] Ilson D.H., van Hillegersberg R. (2018). Management of Patients with Adenocarcinoma or Squamous Cancer of the Esophagus. Gastroenterology.

[4] Botterweck A.A., Schouten L., Volovics A., Dorant E., Brandt P.V.D. (2000). Trends in incidence of adenocarcinoma of the oesophagus and gastric cardia in ten European countries. Leuk. Res..

[5] Desai M., Lieberman D.A., Kennedy K.F., Hamade N., Thota P., Parasa S., Gorrepati V.S., Bansal A., Gupta N., Gaddam S. (2019). Increasing prevalence of high-grade dysplasia and adenocarcinoma on index endoscopy in Barrett’s esophagus over the past 2 decades: Data from a multicenter U.S. consortium. Gastrointest. Endosc..

[6] Pohl H., Welch H.G. (2005). The Role of Overdiagnosis and Reclassification in the Marked Increase of Esophageal Adenocarcinoma Incidence. Gynecol. Oncol..

[7] Sung H., Ferlay J., Siegel R.L., Laversanne M., Soerjomataram I., Jemal A., Bray F. (2021). Global Cancer Statistics 2020: GLOBOCAN Estimates of Incidence and Mortality Worldwide for 36 Cancers in 185 Countries. CA Cancer J. Clin..

[8] Gilbert E.W., Luna R.A., Harrison V.L., Hunter J.G. (2011). Barrett’s Esophagus: A Review of the Literature. J. Gastrointest. Surg..

[9] De Sá I.M., Leal C., Silva J., Falcão D., Felix C., Nascimento C., Carvalho P.B., Vasconcelos H., Pedroto I., Chagas C. (2021). Prevalence of Barrett’s esophagus in a Southern European country: A multicenter study. Eur. J. Gastroenterol. Hepatol..

[10] Ronkainen J., Aro P., Storskrubb T., Johansson S., Lind T., Bolling–Sternevald E., Vieth M., Stolte M., Talley N.J., Agréus L. (2005). Prevalence of Barrett’s Esophagus in the General Population: An Endoscopic Study. Gastroenterology.

[11] Post P.N., Siersema P.D., Van Dekken H. (2007). Rising incidence of clinically evident Barrett’s oesophagus in The Netherlands: A nation-wide registry of pathology reports. Scand. J. Gastroenterol..

[12] Coleman H.G., Bhat S., Murray L.J., McManus D., Gavin A.T., Johnston B.T. (2011). Increasing incidence of Barrett’s oesophagus: A population-based study. Eur. J. Epidemiol..

[13] Muthusamy V.R., Wani S., Gyawali C.P., Komanduri S., Bergman J., Canto M.I., Chak A., Corley D., Falk G.W., Fitzgerald R. (2022). AGA Clinical Practice Update on New Technology and Innovation for Surveillance and Screening in Barrett’s Esophagus: Expert Review. Clin. Gastroenterol. Hepatol..

[14] Cook M.B., Wild C.P., Forman D. (2005). A Systematic Review and Meta-Analysis of the Sex Ratio for Barrett’s Esophagus, Erosive Reflux Disease, and Nonerosive Reflux Disease. Eur. J. Epidemiol..

[15] Thukkani N., Sonnenberg A. (2010). The influence of environmental risk factors in hospitalization for GERD-related diagnoses in the United States. Aliment. Pharmacol. Ther..

[16] A Anderson L., Watson R.P., Murphy S.J., Johnston B.T., Comber H., Mc Guigan J., Reynolds J.V., Murray L.J. (2007). Risk factors for Barrett’s oesophagus and oesophageal adenocarcinoma: Results from the FINBAR study. World J. Gastroenterol..

[17] Andrici J., Cox M.R., Eslick G.D. (2013). Cigarette smoking and the risk of Barrett’s esophagus: A systematic review and meta-analysis. J. Gastroenterol. Hepatol..

[18] Cook M.B., Shaheen N.J., Anderson L.A., Giffen C., Chow W., Vaughan T.L., Whiteman D.C., Corley D.A. (2012). Cigarette Smoking Increases Risk of Barrett’s Esophagus: An Analysis of the Barrett’s and Esophageal Adenocarcinoma Consortium. Gastroenterology.

[19] Eloubeidi M.A., Provenzale D. (2001). Clinical and Demographic Predictors of Barrett’s Esophagus Among Patients with Gastroesophageal Reflux Disease. J. Clin. Gastroenterol..

[20] Corley D.A., Kubo A., Levin T.R., Block G., Habel L., Zhao W., Leighton P., Quesenberry C., Rumore G.J., Buffler P.A. (2007). Abdominal Obesity and Body Mass Index as Risk Factors for Barrett’s Esophagus. Gastroenterology.

[21] Edelstein Z.R., Farrow D.C., Bronner M.P., Rosen S.N., Vaughan T.L. (2007). Central Adiposity and Risk of Barrett’s Esophagus. Gastroenterology.

[22] Mokrowiecka A., Daniel P., Jasińska A., Pietruczuk M., Pawłowski M., Szczesniak P., Orszulak-Michalak D., Malecka-Panas E. (2012). Serum Adiponectin, Resistin, Leptin Concentration and Central Adiposity Parameters in Barrett’s Esophagus Patients with and without Intestinal Metaplasia in Comparison to Healthy Controls and Patients with GERD. Hepato-Gastroenterology.

[23] Chak A., Ochs-Balcom H., Falk G., Grady W.M., Kinnard M., Willis J.E., Elston R., Eng C. (2006). Familiality in Barrett’s Esophagus, Adenocarcinoma of the Esophagus, and Adenocarcinoma of the Gastroesophageal Junction. Cancer Epidemiol. Biomark. Prev..

[24] Juhasz A., Mittal S.K., Lee T.H., Deng C., Chak A., Lynch H.T. (2011). Prevalence of Barrett Esophagus in First-Degree Relatives of Patients with Esophageal Adenocarcinoma. J. Clin. Gastroenterol..

[25] Fock K.M., Talley N., Goh K.L., Sugano K., Katelaris P., Holtmann G., Pandolfino J.E., Sharma P., Ang T.L., Hongo M. (2016). Asia-Pacific consensus on the management of gastro-oesophageal reflux disease: An update focusing on refractory reflux disease and Barrett’s oesophagus. Gut.

[26] Edelstein Z.R., Bronner M.P., Rosen S.N., Vaughan T.L. (2009). Risk Factors for Barrett’s Esophagus Among Patients with Gastroesophageal Reflux Disease: A Community Clinic-Based Case–Control Study. Am. J. Gastroenterol..

[27] Eusebi L.H., Telese A., Cirota G.G., Haidry R., Zagari R.M., Bazzoli F., Ford A.C. (2021). Systematic review with meta-analysis: Risk factors for Barrett’s oesophagus in individuals with gastro-oesophageal reflux symptoms. Aliment. Pharmacol. Ther..

[28] Kambhampati S., Tieu A.H., Luber B., Wang H., Meltzer S.J. (2020). Risk Factors for Progression of Barrett’s Esophagus to High Grade Dysplasia and Esophageal Adenocarcinoma. Sci. Rep..

[29] Rubenstein J.H., McConnell D., Waljee A.K., Metko V., Nofz K., Khodadost M., Jiang L., Raghunathan T. (2020). Validation and Comparison of Tools for Selecting Individuals to Screen for Barrett’s Esophagus and Early Neoplasia. Gastroenterology.

[30] Xie S.-H., Ness-Jensen E., Medefelt N., Lagergren J. (2018). Open: Assessing the Feasibility of Targeted Screening for Esophageal Adenocarcinoma Based on Individual Risk Assessment in a Population-Based Cohort Study in Norway (The HUNT Study). Am. J. Gastroenterol..

[31] Rubenstein J.H., Morgenstern H., Appelman H., Scheiman J., Schoenfeld P., McMahon L.F., Metko V., Near E., Kellenberg J., Kalish T. (2013). Prediction of Barrett’s Esophagus Among Men. Am. J. Gastroenterol..

[32] Kunzmann A.T., Thrift A.P., Cardwell C.R., Lagergren J., Xie S., Johnston B.T., Anderson L., Busby J., McMenamin Ú.C., Spence A.D. (2018). Model for Identifying Individuals at Risk for Esophageal Adenocarcinoma. Clin. Gastroenterol. Hepatol..

[33] Fitzgerald R.C., Di Pietro M., Ragunath K., Ang Y., Kang J.-Y., Watson P., Trudgill N., Patel P., Kaye P.V., Sanders S. (2014). British Society of Gastroenterology guidelines on the diagnosis and management of Barrett’s oesophagus. Gut.

[34] Herrero L.A., Curvers W.L., van Vilsteren F.G.I., Wolfsen H., Ragunath K., Song L.-M.W.K., Mallant-Hent R.C., van Oijen A., Scholten P., Schoon E.J. (2013). Validation of the Prague C&M classification of Barrett’s esophagus in clinical practice. Endoscopy.

[35] Gorrepati V.S., Sharma P. (2018). How Should We Report Endoscopic Results in Patient’s with Barrett’s Esophagus?. Dig. Dis. Sci..

[36] Sharma P., Dent J., Armstrong D., Bergman J.J., Gossner L., Hoshihara Y., Jankowski J., Junghard O., Lundell L., Tytgat G.N. (2006). The Development and Validation of an Endoscopic Grading System for Barrett’s Esophagus: The Prague C & M Criteria. Gastroenterology.

[37] Participants in the Paris Workshop (2003). The Paris endoscopic classification of superficial neoplastic lesions: Esophagus, stomach, and colon: November 30 to December 1, 2002. Gastrointest. Endosc..

[38] Maroni R., Barnes J., Offman J., Scheibl F., Smith S.G., Debiram-Beecham I., Waller J., Sasieni P., Fitzgerald R.C., Rubin G. (2022). Patient-reported experiences and views on the Cytosponge test: A mixed-methods analysis from the BEST3 trial. BMJ Open.

[39] A Katzka D., Smyrk T.C., A Alexander J., Geno D.M., A Beitia R., O Chang A., Shaheen N.J., Fitzgerald R.C., Dellon E.S. (2017). Accuracy and Safety of the Cytosponge for Assessing Histologic Activity in Eosinophilic Esophagitis: A Two-Center Study. Am. J. Gastroenterol..

[40] Benaglia T., Sharples L.D., Fitzgerald R.C., Lyratzopoulos G. (2013). Health Benefits and Cost Effectiveness of Endoscopic and Nonendoscopic Cytosponge Screening for Barrett’s Esophagus. Gastroenterology.

[41] De Jonge P.J.F., Van Blankenstein M., Looman C.W.N., Casparie M.K., A Meijer G., Kuipers E.J. (2010). Risk of malignant progression in patients with Barrett’s oesophagus: A Dutch nationwide cohort study. Gut.

[42] Desai T.K., Krishnan K., Samala N., Singh J., Cluley J., Perla S., Howden C.W. (2012). The incidence of oesophageal adenocarcinoma in non-dysplastic Barrett’s oesophagus: A meta-analysis. Gut.

[43] Sikkema M., de Jonge P.J., Steyerberg E.W., Kuipers E.J. (2010). Risk of Esophageal Adenocarcinoma and Mortality in Patients With Barrett’s Esophagus: A Systematic Review and Meta-analysis. Clin. Gastroenterol. Hepatol..

[44] Thomas T., Abrams K.R., De Caestecker J.S., Robinson R.J. (2007). Meta analysis: Cancer risk in Barrett’s oesophagus. Aliment. Pharmacol. Ther..

[45] Wani S., Puli S.R., Shaheen N.J., Westhoff B., Slehria S., Bansal A., Rastogi A., Sayana H., Sharma P. (2009). Esophageal Adenocarcinoma in Barrett’s Esophagus After Endoscopic Ablative Therapy: A Meta-Analysis and Systematic Review. Am. J. Gastroenterol..

[46] Yousef F., Cardwell C., Cantwell M.M., Galway K., Johnston B.T., Murray L. (2008). The Incidence of Esophageal Cancer and High-Grade Dysplasia in Barrett’s Esophagus: A Systematic Review and Meta-Analysis. Eur. J. Epidemiol..

[47] Shaheen N.J., Crosby M.A., Bozymski E.M., Sandler R.S. (2000). Is there publication bias in the reporting of cancer risk in Barrett’s esophagus?. Gastroenterology.

[48] Hvid-Jensen F., Pedersen L., Drewes A.M., Sørensen H.T., Funch-Jensen P. (2011). Incidence of Adenocarcinoma among Patients with Barrett’s Esophagus. N. Engl. J. Med..

[49] Kunzmann A.T., Coleman H.G., Johnston B.T., Turkington R.C., McManus D., Anderson L., Thrift A.P. (2021). Does Risk of Progression from Barrett’s Esophagus to Esophageal Adenocarcinoma Change Based on the Number of Non-dysplastic Endoscopies?. Dig. Dis. Sci..

[50] Chandrasekar V.T., Hamade N., Desai M., Rai T., Gorrepati V.S., Jegadeesan R., Sathyamurthy A., Sharma P. (2019). Significantly lower annual rates of neoplastic progression in short- compared to long-segment non-dysplastic Barrett’s esophagus: A systematic review and meta-analysis. Endoscopy.

[51] Qumseya B., Sultan S., Bain P., Jamil L., Jacobson B., Anandasabapathy S., Agrawal D., Buxbaum J.L., Fishman D.S., Gurudu S.R. (2019). ASGE guideline on screening and surveillance of Barrett’s esophagus. Gastrointest. Endosc..

[52] Weusten B., Bisschops R., Coron E., Dinis-Ribeiro M., Dumonceau J.-M., Esteban J.-M., Hassan C., Pech O., Repici A., Bergman J. (2017). Endoscopic management of Barrett’s esophagus: European Society of Gastrointestinal Endoscopy (ESGE) Position Statement. Endoscopy.

[53] Corley D.A., Mehtani K., Quesenberry C., Zhao W., de Boer J., Weiss N.S. (2013). Impact of Endoscopic Surveillance on Mortality from Barrett’s Esophagus–Associated Esophageal Adenocarcinomas. Gastroenterology.

[54] Codipilly D.C., Chandar A.K., Singh S., Wani S., Shaheen N.J., Inadomi J.M., Chak A., Iyer P.G. (2018). The Effect of Endoscopic Surveillance in Patients with Barrett’s Esophagus: A Systematic Review and Meta-analysis. Gastroenterology.

[55] Curvers W.L., Kate F.J.T., Krishnadath K.K., Visser M., Elzer B., Baak L.C., Bohmer C., Mallant-Hent R.C., van Oijen A., Naber A.H. (2010). Low-Grade Dysplasia in Barrett’s Esophagus: Overdiagnosed and Underestimated. Am. J. Gastroenterol..

[56] Duits L.C., Phoa K.N., Curvers W.L., Kate F.J.W.T., A Meijer G., A Seldenrijk C., Offerhaus G.J., Visser M., Meijer S.L., Krishnadath K.K. (2015). Barrett’s oesophagus patients with low-grade dysplasia can be accurately risk-stratified after histological review by an expert pathology panel. Gut.

[57] Lim C., Treanor D., Dixon M., Axon A. (2007). Low-grade dysplasia in Barrett’s esophagus has a high risk of progression. Endoscopy.

[58] Thota P.N., Lee H.-J., Goldblum J.R., Liu X., Sanaka M.R., Gohel T., Kanadiya M., Lopez R. (2015). Risk Stratification of Patients with Barrett’s Esophagus and Low-grade Dysplasia or Indefinite for Dysplasia. Clin. Gastroenterol. Hepatol..

[59] Vieth M., Schubert B., Lang-Schwarz K., Stolte M. (2006). Frequency of Barrett’s neoplasia after initial negative endoscopy with biopsy: A long-term histopathological follow-up study. Endoscopy.

[60] Wani S., Falk G., Post J., Yerian L., Hall M., Wang A., Gupta N., Gaddam S., Singh M., Singh V. (2011). Risk Factors for Progression of Low-Grade Dysplasia in Patients with Barrett’s Esophagus. Gastroenterology.

[61] Reid B.J., Levine D.S., Longton G., Blount P.L., Rabinovitch P.S. (2000). Predictors of Progression to Cancer in Barrett’s Esophagus: Baseline Histology and Flow Cytometry Identify Low- and High-Risk Patient Subsets. Am. J. Gastroenterol..

[62] Rice T.W. (1999). Commentary: Esophageal carcinoma confined to the wall—The need for immediate definitive therapy. J. Thorac. Cardiovasc. Surg..

[63] Dunbar K.B., Spechler S.J. (2012). The Risk of Lymph-Node Metastases in Patients with High-Grade Dysplasia or Intramucosal Carcinoma in Barrett’s Esophagus: A Systematic Review. Am. J. Gastroenterol..

[64] Downs-Kelly E., Mendelin J.E., Bennett A.E., A Castilla E., Henricks W.H., Schoenfield L., Skacel M., Yerian L., Rice T.W., Rybicki L.A. (2008). Poor Interobserver Agreement in the Distinction of High-Grade Dysplasia and Adenocarcinoma in Pretreatment Barrett’s Esophagus Biopsies. Am. J. Gastroenterol..

[65] Konda V.J.A., Souza R.F. (2018). Biomarkers of Barrett’s Esophagus: From the Laboratory to Clinical Practice. Dig. Dis. Sci..

[66] Mokrowiecka A., Wierzchniewska-Ławska A., Smolarz B., Romanowicz-Makowska H., Malecka-Panas E. (2012). p16 gene mutations in Barrett’s esophagus in gastric metaplasia—intestinal metaplasia—dysplasia—adenocarcinoma sequence. Adv. Med. Sci..

[67] Stachler M.D., Taylor-Weiner A., Peng S., McKenna A., Agoston A.T., Odze R.D., Davison J.M., Nason K.S., Loda M., Leshchiner I. (2015). Paired exome analysis of Barrett’s esophagus and adenocarcinoma. Nat. Genet..

[68] Kastelein F., Biermann K., Steyerberg E.W., Verheij J., Kalisvaart M., Looijenga L.H.J., A Stoop H., Walter L., Kuipers E.J., Spaander M.C.W. (2013). Aberrant p53 protein expression is associated with an increased risk of neoplastic progression in patients with Barrett’s oesophagus. Gut.

[69] Mokrowiecka A., Wierzchniewska-Ławska A., Smolarz B., Romanowicz-Makowska H., Malecka-Panas E. (2009). Polymorphism/loss of heterozygosity of APC gene in GERD-Barrett’s metaplasia-dysplasia-adenocarcinoma sequence. Pol. Merkur. Lekarski..

[70] Gupta N., Gaddam S., Wani S.B., Bansal A., Rastogi A., Sharma P. (2012). Longer inspection time is associated with increased detection of high-grade dysplasia and esophageal adenocarcinoma in Barrett’s esophagus. Gastrointest. Endosc..

[71] Connor M.J., Sharma P. (2004). Chromoendoscopy and magnification endoscopy for diagnosing esophageal cancer and dysplasia. Thorac. Surg. Clin..

[72] Horwhat J.D., Maydonovitch C.L., Ramos F., Colina R., Gaertner E., Lee H., Wong R.K. (2008). A Randomized Comparison of Methylene Blue-Directed Biopsy Versus Conventional Four-Quadrant Biopsy for the Detection of Intestinal Metaplasia and Dysplasia in Patients with Long-Segment Barrett’s Esophagus. Am. J. Gastroenterol..

[73] Lim C.H., Rotimi O., Dexter S.P., Axon A.T. (2006). Randomized crossover study that used methylene blue or random 4-quadrant biopsy for the diagnosis of dysplasia in Barrett’s esophagus. Gastrointest. Endosc..

[74] Ngamruengphong S., Sharma V.K., Das A. (2009). Diagnostic yield of methylene blue chromoendoscopy for detecting specialized intestinal metaplasia and dysplasia in Barrett’s esophagus: A meta-analysis. Gastrointest. Endosc..

[75] Olliver J., Wild C., Sahay P., Dexter S., Hardie L. (2003). Chromoendoscopy with methylene blue and associated DNA damage in Barrett’s oesophagus. Lancet.

[76] Repici A., Ciscato C., Wallace M., Sharma P., Anderloni A., Carrara S., Di Leo M., Hassan C. (2018). Evaluation of genotoxicity related to oral methylene blue chromoendoscopy. Endoscopy.

[77] Coletta M., Sami S.S., Nachiappan A., Fraquelli M., Casazza G., Ragunath K. (2016). Acetic acid chromoendoscopy for the diagnosis of early neoplasia and specialized intestinal metaplasia in Barrett’s esophagus: A meta-analysis. Gastrointest. Endosc..

[78] Bhandari P., Kandaswamy P., Cowlishaw D., Longcroft-Wheaton G. (2012). Acetic acid-enhanced chromoendoscopy is more cost-effective than protocol-guided biopsies in a high-risk Barrett’s population. Dis. Esophagus.

[79] Dos Santos C.E.O., Lima J.C.P., Lopes C.V., Malaman D., Salomão A.D., Garcia A.C., Teixeira C.R. (2010). Computerized virtual chromoendoscopy versus indigo carmine chromoendoscopy combined with magnification for diagnosis of small colorectal lesions. Eur. J. Gastroenterol. Hepatol..

[80] Gono K., Obi T., Yamaguchi M., Ohyama N., Machida H., Sano Y., Yoshida S., Hamamoto Y., Endo T. (2004). Appearance of enhanced tissue features in narrow-band endoscopic imaging. J. Biomed. Opt..

[81] Sharma P., Hawes R.H., Bansal A., Gupta N., Curvers W., Rastogi A., Singh M., Hall M., Mathur S.C., Wani S.B. (2013). Standard endoscopy with random biopsies versus narrow band imaging targeted biopsies in Barrett’s oesophagus: A prospective, international, randomised controlled trial. Gut.

[82] Furneri G., Klausnitzer R., Haycock L., Ihara Z. (2019). Economic value of narrow-band imaging versus white light endoscopy for the diagnosis and surveillance of Barrett’s esophagus: Cost-consequence model. PLoS ONE.

[83] Picot J., Rose M., Cooper K., Pickett K., Lord J., Harris P., Whyte S., Böhning D., Shepherd J. (2017). Virtual chromoendoscopy for the real-time assessment of colorectal polyps in vivo: A systematic review and economic evaluation. Health Technol. Assess..

[84] Hoffman A., Korczynski O., Tresch A., Hansen T., Rahman F., Goetz M., Murthy S., Galle P.R., Kiesslich R. (2014). Acetic acid compared with i-scan imaging for detecting Barrett’s esophagus: A randomized, comparative trial. Gastrointest. Endosc..

[85] Bruno M.J. (2003). Magnification endoscopy, high resolution endoscopy, and chromoscopy; towards a better optical diagnosis. Gut.

[86] Wasielica-Berger J., Baniukiewicz A., Wroblewski E., Chwiesko A., Dabrowski A. (2011). Magnification Endoscopy and Chromoendoscopy in Evaluation of Specialized Intestinal Metaplasia in Barrett’s Esophagus. Dig. Dis. Sci..

[87] Endo T., Awakawa T., Takahashi H., Arimura Y., Itoh F., Yamashita K., Sasaki S., Yamamoto H., Tang X., Imai K. (2002). Classification of Barrett’s epithelium by magnifying endoscopy. Gastrointest. Endosc..

[88] Nguyen T.H., Thrift A.P., Rugge M., El-Serag H.B. (2021). Prevalence of Barrett’s esophagus and performance of societal screening guidelines in an unreferred primary care population of U.S. veterans. Gastrointest. Endosc..

[89] Sawas T., Zamani S.A., Killcoyne S., Dullea A., Wang K.K., Iyer P.G., Fitzgerald R.C., Katzka D.A. (2022). Limitations of Heartburn and Other Societies’ Criteria in Barrett’s Screening for Detecting De Novo Esophageal Adenocarcinoma. Clin. Gastroenterol. Hepatol..

[90] El-Serag H.B., Sweet S., Winchester C.C., Dent J. (2014). Update on the epidemiology of gastro-oesophageal reflux disease: A systematic review. Gut.

[91] Shaheen N.J., Falk G.W., Iyer P.G., Souza R.F., Yadlapati R.H., Sauer B.G., Wani S. (2022). Diagnosis and Management of Barrett’s Esophagus: An Updated ACG Guideline. Am. J. Gastroenterol..

[92] Bennett C., Moayyedi P., A Corley D., de Caestecker J., Falck-Ytter Y., Falk G., Vakil N., Sanders S., Vieth M., Inadomi J. (2015). BOB CAT: A Large-Scale Review and Delphi Consensus for Management of Barrett’s Esophagus with No Dysplasia, Indefinite for, or Low-Grade Dysplasia. Am. J. Gastroenterol..

[93] Ratcliffe E.G., Jankowski J.A. (2019). Gastroesophageal reflux disease and Barrett esophagus: An overview of evidence-based guidelines. Pol. Arch. Intern. Med..

[94] E Verbeek R., Leenders M., Kate F.J.W.T., van Hillegersberg R., Vleggaar F.P., van Baal J.W.P.M., van Oijen M.G.H., Siersema P.D. (2014). Surveillance of Barrett’s Esophagus and Mortality from Esophageal Adenocarcinoma: A Population-Based Cohort Study. Am. J. Gastroenterol..

[95] Inadomi J.M., Saxena N. (2018). Screening and Surveillance for Barrett’s Esophagus: Is It Cost-Effective?. Dig. Dis. Sci..

[96] Lee S.-W., Lien H.-C., Chang C.-S., Lin M.-X., Chang C.-H., Ko C.-W. (2018). Benefits of the Seattle biopsy protocol in the diagnosis of Barrett’s esophagus in a Chinese population. World J. Clin. Cases.

[97] Nachiappan A., Ragunath K., Card T., Kaye P. (2020). Diagnosing dysplasia in Barrett’s oesophagus still requires Seattle protocol biopsy in the era of modern video endoscopy: Results from a tertiary centre Barrett’s dysplasia database. Scand. J. Gastroenterol..

[98] Visrodia K., Singh S., Krishnamoorthi R., Ahlquist D.A., Wang K.K., Iyer P.G., Katzka D.A. (2016). Magnitude of Missed Esophageal Adenocarcinoma After Barrett’s Esophagus Diagnosis: A Systematic Review and Meta-analysis. Gastroenterology.

[99] Agha Y.H., Srinivasan S., Hyder J., Wuthnow C., Taleb A., Tofteland N., Kilgore W., Salyers W. (2021). WATS(3D) versus forceps biopsy in screening for Barrett’s esophagus: Experience in community endoscopy centers. Ann. Gastroenterol..

[100] Pan W., Li X., Wang W., Zhou L., Wu J., Ren T., Liu C., Lv M., Su S., Tang Y. (2021). Identification of Barrett’s esophagus in endoscopic images using deep learning. BMC Gastroenterol..

[101] Tan J.L., Chinnaratha M.A., Woodman R., Martin R., Chen H.-T., Carneiro G., Singh R. (2022). Diagnostic Accuracy of Artificial Intelligence (AI) to Detect Early Neoplasia in Barrett’s Esophagus: A Non-comparative Systematic Review and Meta-Analysis. Front. Med..

[102] Enestvedt B.K., Lugo R., Guarner-Argente C., Shah P., Falk G., Furth E., Ginsberg G.G. (2013). Location, location, location: Does early cancer in Barrett’s esophagus have a preference?. Gastrointest. Endosc..

[103] Krajciova J., Vackova Z., Spicak J., Martinek J. (2018). Radiofrequency ablation for Barrett’s esophagus-related neoplasia. Int. J. Clin. Rev..

[104] Di Pietro M., Fitzgerald R.C. (2018). Revised British Society of Gastroenterology recommendation on the diagnosis and management of Barrett’s oesophagus with low-grade dysplasia. Gut.

[105] Qumseya B.J., Wani S., Gendy S., Harnke B., Bergman J.J., Wolfsen H. (2017). Disease Progression in Barrett’s Low-Grade Dysplasia With Radiofrequency Ablation Compared With Surveillance: Systematic Review and Meta-Analysis. Am. J. Gastroenterol..

[106] Klair J.S., Zafar Y., Nagra N., Murali A.R., Jayaraj M., Singh D., Rustagi T., Krishnamoorthi R. (2021). Outcomes of Radiofrequency Ablation versus Endoscopic Surveillance for Barrett’s Esophagus with Low-Grade Dysplasia: A Systematic Review and Meta-Analysis. Dig. Dis..

[107] Kahn A., Al-Qaisi M., Temkit M., Kommineni V.T., Callaway J.K., Boroff E.S., Burdick G.E., Lam-Himlin D.M., Vela M.F., Ramirez F.C. (2018). Longitudinal outcomes of radiofrequency ablation versus surveillance endoscopy for Barrett’s esophagus with low-grade dysplasia. Dis. Esophagus.

[108] Shaheen N.J., Sharma P., Overholt B.F., Wolfsen H.C., Sampliner R.E., Wang K.K., Galanko J.A., Bronner M.P., Goldblum J.R., Bennett A.E. (2009). Radiofrequency Ablation in Barrett’s Esophagus with Dysplasia. N. Engl. J. Med..

[109] Manner H., Rabenstein T., Pech O., Braun K., May A., Pohl J., Behrens A., Vieth M., Ell C. (2014). Ablation of residual Barrett’s epithelium after endoscopic resection: A randomized long-term follow-up study of argon plasma coagulation vs. surveillance (APE study). Endoscopy.

[110] Sharma P., Shaheen N.J., Katzka D., Bergman J.J. (2020). AGA Clinical Practice Update on Endoscopic Treatment of Barrett’s Esophagus with Dysplasia and/or Early Cancer: Expert Review. Gastroenterology.

[111] Whiteman D.C., Appleyard M., Bahin F.F., Bobryshev Y.V., Bourke M.J., Brown I., Chung A., Clouston A., Dickins E., Emery J. (2015). Australian clinical practice guidelines for the diagnosis and management of Barrett’s esophagus and early esophageal adenocarcinoma. J. Gastroenterol. Hepatol..

[112] Hu Y., Puri V., Shami V.M., Stukenborg G.J., Kozower B.D. (2016). Comparative Effectiveness of Esophagectomy Versus Endoscopic Treatment for Esophageal High-grade Dysplasia. Ann. Surg..

[113] Chu J.N., Choi J., Tramontano A., Morse C., Forcione D., Nishioka N.S., Abrams J.A., Rubenstein J.H., Kong C.Y., Inadomi J.M. (2018). Surgical vs Endoscopic Management of T1 Esophageal Adenocarcinoma: A Modeling Decision Analysis. Clin. Gastroenterol. Hepatol..

[114] Wani S., Drahos J., Cook M.B., Rastogi A., Bansal A., Yen R., Sharma P., Das A. (2014). Comparison of endoscopic therapies and surgical resection in patients with early esophageal cancer: A population-based study. Gastrointest. Endosc..

[115] Holmes I., Hing T., Friedland S. (2016). Combining endoscopic submucosal dissection and endoscopic mucosal resection to treat neoplasia in Barrett’s esophagus. Surg. Endosc..

[116] Ishihara R., Iishi H., Uedo N., Takeuchi Y., Yamamoto S., Yamada T., Masuda E., Higashino K., Kato M., Narahara H. (2008). Comparison of EMR and endoscopic submucosal dissection for en bloc resection of early esophageal cancers in Japan. Gastrointest. Endosc..

[117] Pouw R.E., van Vilsteren F.G., Peters F.P., Herrero L.A., Kate F.J.T., Visser M., Schenk B.E., Schoon E.J., Peters F.T., Houben M. (2011). Randomized trial on endoscopic resection-cap versus multiband mucosectomy for piecemeal endoscopic resection of early Barrett’s neoplasia. Gastrointest. Endosc..

[118] Oka S., Tanaka S., Saito Y., Iishi H., Kudo S.-E., Ikematsu H., Igarashi M., Saitoh Y., Inoue Y., Kobayashi K. (2015). Local Recurrence After Endoscopic Resection for Large Colorectal Neoplasia: A Multicenter Prospective Study in Japan. Am. J. Gastroenterol..

[119] Geramizadeh B., Owen D.A. (2017). Handling and Pathology Reporting of Gastrointestinal Endoscopic Mucosal Resection. Middle East J. Dig. Dis..

[120] Nishizawa T., Yahagi N. (2017). Endoscopic mucosal resection and endoscopic submucosal dissection. Curr. Opin. Gastroenterol..

[121] Pimentel-Nunes P., Libânio D., Bastiaansen B.A.J., Bhandari P., Bisschops R., Bourke M.J., Esposito G., Lemmers A., Maselli R., Messmann H. (2022). Endoscopic submucosal dissection for superficial gastrointestinal lesions: European Society of Gastrointestinal Endoscopy (ESGE) Guideline—Update 2022. Endoscopy.

[122] Sun F., Yuan P., Chen T., Hu J. (2014). Efficacy and complication of endoscopic submucosal dissection for superficial esophageal carcinoma: A systematic review and meta-analysis. J. Cardiothorac. Surg..

[123] Guo H.-M. (2014). Endoscopic submucosal dissection vs endoscopic mucosal resection for superficial esophageal cancer. World J. Gastroenterol..

[124] Draganov P.V., Wang A.Y., Othman M.O., Fukami N. (2019). AGA Institute Clinical Practice Update: Endoscopic Submucosal Dissection in the United States. Clin. Gastroenterol. Hepatol..

[125] Gurwara S., Clayton S. (2019). Esophageal Perforations: An Endoscopic Approach to Management. Curr. Gastroenterol. Rep..

[126] Eroğlu A., Aydın Y., Yılmaz Ö. (2018). Minimally invasive management of esophageal perforation. Turk. J. Thorac. Cardiovasc. Surg..

[127] Van Munster S., Nieuwenhuis E., Weusten B.L.A.M., Herrero L.A., Bogte A., Alkhalaf A., E Schenk B., Schoon E.J., Curvers W., Koch A.D. (2022). Long-term outcomes after endoscopic treatment for Barrett’s neoplasia with radiofrequency ablation ± endoscopic resection: Results from the national Dutch database in a 10-year period. Gut.

[128] Kahn A., Shaheen N.J., Iyer P.G. (2020). Approach to the Post-Ablation Barrett’s Esophagus Patient. Am. J. Gastroenterol..

[129] Yang L.S., Holt B.A., Williams R., Norris R., Tsoi E., Cameron G., Desmond P., Taylor A.C. (2021). Endoscopic features of buried Barrett’s mucosa. Gastrointest. Endosc..

[130] Konda V., Souza R.F., Dunbar K.B., Mills J.C., Kim D.S., Odze R.D., Spechler S.J. (2022). An Endoscopic and Histologic Study on Healing of Radiofrequency Ablation Wounds in Patients with Barrett’s Esophagus. Am. J. Gastroenterol..

[131] Van Hagen P., Hulshof M.C.C.M., Van Lanschot J.J.B., Steyerberg E.W., van Berge Henegouwen M.I., Wijnhoven B.P.L., Richel D.J., Nieuwenhuijzen G.A.P., Hospers G.A.P., Bonenkamp J.J. (2012). Preoperative Chemoradiotherapy for Esophageal or Junctional Cancer. N. Engl. J. Med..

[132] Morgan M.A., Lewis W.G., Casbard A., Roberts S.A., Adams R., Clark G.W.B., Havard T.J., Crosby T.D.L. (2009). Stage-for-stage comparison of definitive chemoradiotherapy, surgery alone and neoadjuvant chemotherapy for oesophageal carcinoma. Br. J. Surg..

[133] Sun L., Zhang Z., Xu J., Xu G., Liu X. (2017). Dietary fiber intake reduces risk for Barrett’s esophagus and esophageal cancer. Crit. Rev. Food Sci. Nutr..

[134] Coleman H.G., Murray L.J., Hicks B., Bhat S.K., Kubo A., Corley D.A., Cardwell C., Cantwell M.M. (2013). Dietary fiber and the risk of precancerous lesions and cancer of the esophagus: A systematic review and meta-analysis. Nutr. Rev..

[135] McFadden D.W., Riggs D.R., Jackson B.J., Cunningham C. (2008). Corn-derived carbohydrate inositol hexaphosphate inhibits Barrett’s adenocarcinoma growth by pro-apoptotic mechanisms. Oncol. Rep..

[136] Morozov S., Isakov V., Konovalova M. (2018). Fiber-enriched diet helps to control symptoms and improves esophageal motility in patients with non-erosive gastroesophageal reflux disease. World J. Gastroenterol..

[137] Zhao Z., Yin Z., Zhang C. (2021). Lifestyle interventions can reduce the risk of Barrett’s esophagus: A systematic review and meta-analysis of 62 studies involving 250,157 participants. Cancer Med..

[138] Jarosz M., Taraszewska A. (2014). Risk factors for gastroesophageal reflux disease—the role of diet. Gastroenterol. Rev..

[139] Zhao Z., Pu Z., Yin Z., Yu P., Hao Y., Wang Q., Guo M., Zhao Q. (2016). Dietary fruit, vegetable, fat and red and processed meat intakes and Barrett’s esophagus risk: A systematic review and meta-analysis. Sci. Rep..

[140] Brandt P.A.V.D. (2022). The impact of a healthy lifestyle on the risk of esophageal and gastric cancer subtypes. Eur. J. Epidemiol..

[141] Li W., Park Y., Wu J.W., Ren J., Goldstein A.M., Taylor P.R., Hollenbeck A.R., Freedman N.D., Abnet C. (2013). Index-based Dietary Patterns and Risk of Esophageal and Gastric Cancer in a Large Cohort Study. Clin. Gastroenterol. Hepatol..

[142] Schulpen M., Peeters P.H., Brandt P.A.V.D. (2019). Mediterranean diet adherence and risk of esophageal and gastric cancer subtypes in the Netherlands Cohort Study. Gastric Cancer.

[143] Balasubramanian G., Gupta N., Giacchino M., Singh M., Kanakadandi V., Gaddam S., Wani S.B., Higbee A.D., Rastogi A., Bansal A. (2013). Cigarette smoking is a modifiable risk factor for Barrett’s oesophagus. United Eur. Gastroenterol. J..

[144] Thrift A.P., Cook M.B., Vaughan T.L., Anderson L., Murray L.J., Whiteman D., Shaheen N.J., A Corley D. (2014). Alcohol and the Risk of Barrett’s Esophagus: A Pooled Analysis from the International BEACON Consortium. Am. J. Gastroenterol..

[145] Xu Q., Guo W., Shi X., Zhang W., Zhang T., Wu C., Lu J., Wang R., Zhao Y., Ma X. (2015). Association Between Alcohol Consumption and the Risk of Barrett’s Esophagus. Medicine.

[146] Lou Z., Xing H., Li D. (2014). Alcohol Consumption and the Neoplastic Progression in Barrett’s Esophagus: A Systematic Review and Meta-Analysis. PLoS ONE.

[147] Chen Y., Sun C., Wu Y., Chen X., Kailas S., Karadsheh Z., Li G., Guo Z., Yang H., Hu L. (2021). Do proton pump inhibitors prevent Barrett’s esophagus progression to high-grade dysplasia and esophageal adenocarcinoma? An updated meta-analysis. J. Cancer Res. Clin. Oncol..

[148] Jankowski J.A.Z., de Caestecker J., Love S.B., Reilly G., Watson P., Sanders S., Ang Y., Morris D., Bhandari P., Brooks C. (2018). Esomeprazole and aspirin in Barrett’s oesophagus (AspECT): A randomised factorial trial. Lancet.

[149] Targownik L.E., Fisher D.A., Saini S.D. (2022). AGA Clinical Practice Update on De-Prescribing of Proton Pump Inhibitors: Expert Review. Gastroenterology.

[150] Singh S., Garg S.K., Singh P.P., Iyer P.G., El-Serag H.B. (2014). Acid-suppressive medications and risk of oesophageal adenocarcinoma in patients with Barrett’s oesophagus: A systematic review and meta-analysis. Gut.

[151] Rayner C.J., Gatenby P. (2016). Effect of antireflux surgery for Barrett’s esophagus: Long-term results. Minerva Chir..

[152] Gatenby P.A.C., Ramus J.R., Caygill C.P.J., Charlett A., Winslet M.C., Watson A. (2009). Treatment modality and risk of development of dysplasia and adenocarcinoma in columnar-lined esophagus. Dis. Esophagus.

[153] Schneider J.L., Zhao W.K., Corley D.A. (2015). Aspirin and Nonsteroidal Anti-Inflammatory Drug Use and the Risk of Barrett’s Esophagus. Dig. Dis. Sci..

[154] Zhang J., Wu H., Wang R. (2021). Effect of nonsteroidal anti-inflammatory drugs on Barrett’s esophagus risk: A systematic review and meta-analysis. Clin. Res. Hepatol. Gastroenterol..

[155] Wang F., Lv Z.S., Fu Y.K. (2011). Nonsteroidal anti-inflammatory drugs and esophageal inflammation—Barrett’s esophagus—adenocarcinoma sequence: A meta-analysis. Dis. Esophagus.

[156] Thrift A.P., Anderson L., Murray L.J., Cook M.B., Shaheen N.J., Rubenstein J.H., El-Serag H.B., Vaughan T.L., Schneider J.L., Whiteman D. (2016). Nonsteroidal Anti-Inflammatory Drug Use is Not Associated with Reduced Risk of Barrett’s Esophagus. Am. J. Gastroenterol..

[157] Khalaf N., Nguyen T., Ramsey D., El–Serag H.B. (2014). Nonsteroidal Anti-inflammatory Drugs and the Risk of Barrett’s Esophagus. Clin. Gastroenterol. Hepatol..

[158] Greer K.B., Kresak A., Bednarchik B., Dawson D.W., Li L., Chak A., Willis J. (2013). Insulin/Insulin-Like Growth Factor-1 Pathway in Barrett’s Carcinogenesis. Clin. Transl. Gastroenterol..

[159] Trowbridge R., Mittal S.K., Sharma P., Hunter W.J., Agrawal D.K. (2012). Vitamin D receptor expression in the mucosal tissue at the gastroesophageal junction. Exp. Mol. Pathol..

[160] Deeb K.K., Trump D.L., Johnson C.S. (2007). Vitamin D signalling pathways in cancer: Potential for anticancer therapeutics. Nat. Rev. Cancer.

[161] Carlberg C., Muñoz A. (2022). An update on vitamin D signaling and cancer. Semin. Cancer Biol..

[162] Rouphael C., Kamal A., Sanaka M.R., Thota P.N. (2018). Vitamin D in esophageal cancer: Is there a role for chemoprevention?. World J. Gastrointest. Oncol..

[163] Dong J., Gharahkhani P., Chow W.-H., Gammon M.D., Liu G., Caldas C., Wu A.H., Ye W., Onstad L., Anderson L.A. (2019). No Association Between Vitamin D Status and Risk of Barrett’s Esophagus or Esophageal Adenocarcinoma: A Mendelian Randomization Study. Clin. Gastroenterol. Hepatol..

[164] Zhang Q., Agoston A.T., Pham T.H., Zhang W., Zhang X., Huo X., Peng S., Bajpai M., Das K., Odze R.D. (2019). Acidic Bile Salts Induce Epithelial to Mesenchymal Transition via VEGF Signaling in Non-Neoplastic Barrett’s Cells. Gastroenterology.

